# Optimizing Cancer Care Delivery through Implementation Science

**DOI:** 10.3389/fonc.2016.00001

**Published:** 2016-01-28

**Authors:** Taiwo Adesoye, Caprice C. Greenberg, Heather B. Neuman

**Affiliations:** ^1^Department of Surgery, University of Wisconsin School of Medicine and Public Health, Madison, WI, USA

**Keywords:** implementation science, dissemination and implementation research, cancer care delivery, breast cancer, knowledge-to-action, decision aid

## Abstract

The 2013 Institute of Medicine report investigating cancer care concluded that the cancer care delivery system is in crisis due to an increased demand for care, increasing complexity of treatment, decreasing work force, and rising costs. Engaging patients and incorporating evidence-based care into routine clinical practice are essential components of a high-quality cancer delivery system. However, a gap currently exists between the identification of beneficial research findings and the application in clinical practice. Implementation research strives to address this gap. In this review, we discuss key components of high-quality implementation research. We then apply these concepts to a current cancer care delivery challenge in women’s health, specifically the implementation of a surgery decision aid for women newly diagnosed with breast cancer.

## Introduction

**Figure 1:**
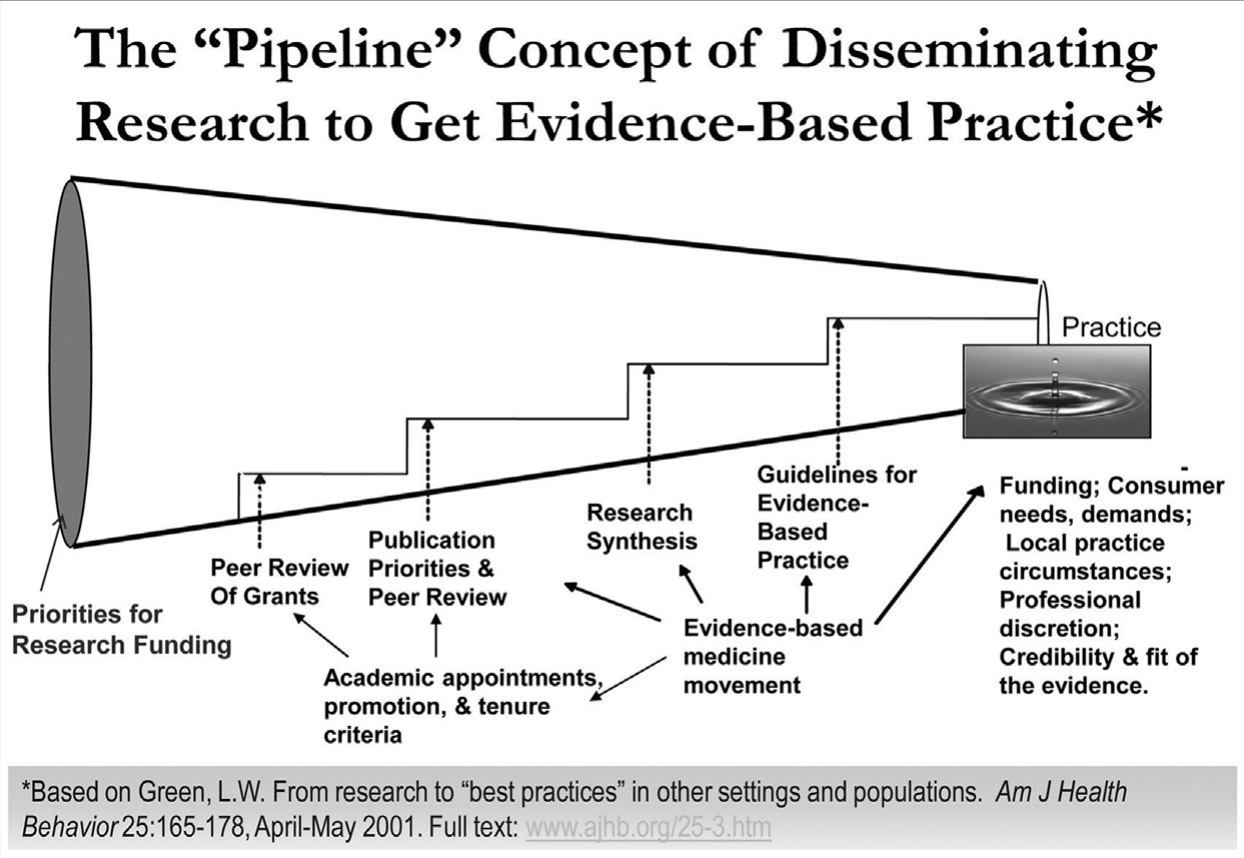
**The pipeline of production and translation of knowledge generated from research into routine clinical practice includes a series of successive screens designed to assure that high-quality research products are delivered to end users**. However, this process results in only 14% of original research being integrated into routine clinical practice and does little to assure that the research products are relevant and/or useful to end users. From Green (5) with permission.

The 2013 Institute of Medicine report investigating cancer care concluded that the cancer care delivery system is in crisis due to an increased demand for care, increasing complexity of treatment, decreasing work force, and rising costs (1). The proposed conceptual framework for a high-quality cancer delivery system highlights the importance of engaging patients and their families, providing evidence-based care, and translating the evidence into routine clinical care. In the current system, translating beneficial research findings to the real world health-care setting is often slow and haphazard despite the proven benefits (2, 3). It has been suggested that an average of 17 years elapses before 14% of original research is integrated into routine physician practice (Figure 1) (4). This gap between the identification of beneficial research findings and the application in clinical practice has led to an increased focus on the processes for implementing new knowledge and the rapidly growing field of dissemination and implementation (D&I) science (5–9). Eccles and Mittman defined implementation research as “the scientific study of methods to promote the systematic uptake of research findings and other evidence-based practices into routine practice” (10). Implementation research spans *implementation* (“the use of strategies to adopt and integrate evidence-based health interventions and change practice patterns within specific settings”) and *dissemination* (“the targeted distribution of information and intervention materials to a specific public health or clinical practice audience”) (11). From past experiences, it is clear that those traditional, passive modes of implementing and disseminating evidence-based practices, such as publication in journals and development of consensus statements, are generally ineffective in sustainably integrating research findings into routine practice (5, 12). Therefore, systematic efforts to identify active, theory-driven implementation strategies are essential (4, 13–17).

## Theoretical Frameworks

Early implementation research was largely “trial and error” with only a minority (10%) of studies providing a theoretical rationale for their approach (18). The absence of a theoretical framework supporting early implementation efforts combined with lack of common terminology to describe processes made it difficult to predict the success of an implementation approach or for others to reproduce the process in other settings (19). A theory-driven approach to implementation that explores explicitly the link between an intervention and an outcome, and systematically strives to explain why the intervention worked or failed in a particular setting is critical to understand and operationalize the key implementation steps (7, 18, 19). In addition to facilitating the implementation for a specific intervention, this type of systematic approach will lead to the creation of generalizable knowledge surrounding methods for the sustainable implementation of an intervention across studies and settings. Theoretical models that broadly inform implementation research are multidisciplinary, pulling from the fields of medicine, public health, psychology, marketing, political science, and even agriculture. In 2011, more than 60 models to support D&I research had been utilized in the literature (20). Considerable effort has been made to consolidate these theories and models to provide researchers with a guide in identifying conceptual models that would best support their work. For example, Tabak et al categorized the theories and models relevant to D&I research according to their focus on dissemination and/or implementation activities and the socio-ecological level to which they are applicable (20). They also rated the flexibility of the model constructs, ranging from a score of 1 (very loose construct definition allowing researchers maximal flexibility in applying the model) to 5 (more defined constructs providing researchers with a more operational, step-by-step approach to D&I research activities). Examples of these categorizations for some commonly used models are presented in Table 1.

**Table 1:** **Categorization of commonly used dissemination and implementation models [adapted from Tabak et al. (20)]**.

	Dissemination and/or implementation	Construct flexibility: loosely defined to highly structured constructs (scale 1–5)	Socio-ecological level				
			System	Community	Organization	Individual	Policy
RE-AIM (8)	D = I	4		X	X	X	
Consolidated framework for implementation research (21)	I-only	4		X	X		
Framework for knowledge translation (22)	D-only	5		X	X	X	
Normalization process theory (23)	I-only	3	X	X	X	X	
Health promotion research center framework (24)	D > I	4		X	X	X	X
The precede–proceed model (25)	D = I	5		X	X	X	
Replicating effective programs plus framework (26)	I-only	4		X	X		

*D, dissemination; I, implementation*.

Two often used frameworks to guide implementation efforts include the consolidated framework for implementation research (CFIR) (21) and the RE-AIM framework (8). The CFIR focuses primarily on implementation. It synthesizes existing constructs from multiple published implementation theories into an overarching typology that can be used to conduct a diagnostic assessment of the implementation and context, track the progress of implementation, and explain the success (or lack of success) of an implementation strategy (21). Included constructs focus on the *characteristics of the intervention*, such as its source, complexity, or cost; *the outer setting*, such as relevant governmental policies and regulations or external pressure from competing organizations; *the inner setting*, such as structural characteristics of an organization, organizational culture, and organization readiness for implementation; the *characteristics of involved individuals*, such as their knowledge and beliefs about an intervention and their belief in their ability to implement the intervention; and *the process of implementation*, including planning the implementation, engaging key individuals, and evaluating the implementation efforts. Researchers can select relevant constructs from this framework to guide assessment of their intervention and monitor implementation progress. By contrast, the RE-AIM framework is an evaluation framework with an equal focus on implementation and dissemination (8). It guides evaluation of the *R*each of an intervention (is the intervention getting to the target population), *E*ffectiveness (is the intervention effective in the real world setting), *A*doption (are target groups adopting the intervention), *l*mplementation [what is the fidelity, i.e., the degree to which the intervention is implemented as originally intended (9)], and *M*aintenance or sustainability (are the effects of the intervention maintained over time) (8). This type of evaluation framework can then facilitate comparisons between different interventions and methods of implementation and can inform both the choice of intervention and the needed implementation strategies.

## Selection of Implementation Strategies

Dissemination and implementation theoretical models provide a systematic approach to developing and evaluating the implementation of interventions. Within these frameworks, specific implementation strategies can be selected that match the needs of a clinical program or practice (16, 17). These strategies vary in nature and complexity from a single component (such as reminders, educational meetings) to multifaceted designs, which include multiple discrete or interwoven strategies (5, 16, 17). Compilations of strategies and specific definitions of each strategy have been created to provide researchers with a mechanism for the identification of important and feasible options to meet the needs of their study. Using concept mapping in a multi-stage project known as the expert recommendations for implementing change (ERIC), Waltz et al. grouped 73 implementation strategies into 9 main clusters with similar conceptual backgrounds (Table 2) (17). The importance and feasibility of each strategy were then rated by experts in the field of implementation science. This type of compilation allows researchers to compare and prioritize different strategies most likely to be successful in their clinical context. Although further work must be done to examine the validity of these groupings, this represents an important resource for researchers developing and implementing interventions.

**Table 2:** **Implementation strategies organized by cluster by Waltz et al. showing mean importance and feasibility ratings provided by a panel of implementation science and clinical experts**.

Implementation strategy cluster	Importance	Feasibility	Example of a strategy rated as both important and feasible
Use evaluative and iterative strategies	4.19	4.01	Provide audit and feedback
Provide interactive assistance	3.67	3.29	Facilitation
Adapt and tailor to context	3.59	3.30	Tailor implementation strategies
Develop stakeholder interrelationships	3.47	3.64	Inform local opinion leaders
Train and educate stakeholders	3.43	3.93	Conduct educational meetings
Support clinicians	3.23	3.06	Facilitate relay of clinical data to providers
Engage consumers	3.25	2.95	Involve patients/consumers and family members
Utilize financial strategies	2.86	2.09	^a^
Change infrastructure	2.40	2.01	^a^

*The importance rating scale ranged from 1 (relatively unimportant) to 5 (extremely important), and the feasibility scale ranged from 1 (not at all feasible) to 5 (extremely feasible)*.

*^a^No implementation strategies in these clusters were rated to be both important and feasible*.

When considering implementation strategies, it is critical to consider the context in which an intervention will be implemented. The real world clinical environment is subjected to contextual factors, unlike the controlled research settings in which evidence-based interventions are often designed and tested (27–30). Contextual factors influence the success of implementation and strategies may need to be modified or additional strategies added to address the unique needs of local sites. These factors are recognized at different levels of the implementation process, such as the individual level, including team interactions and individual skill sets, and the organizational level, where available resources and degree of managerial support for a particular intervention may vary between sites (29). Utilizing active, multifaceted implementation strategies in a manner that considers the local context and aligns with organizational priorities increases the potential of efforts being successful.

## Reporting Intervention Implementation

To ensure adequate description of intervention implementation, a number of guidelines for specifying and reporting details of interventions and the implementation processes used have been created (Table 3) (19, 31–38). The goal of these initiatives was to increase the ability of others to deliver an intervention as originally intended, resulting in better fidelity and potentially leading to improved outcomes. Included as a requirement in many of these guidelines are details of not only the intervention itself but also the implementation process such as descriptions of who administered the intervention, the mode of intervention delivery, how the intervention’s implementation may have been adapted to the local context, and how fidelity to the original intervention was maintained (31–33, 36–38). It is important to also describe the context in which an intervention was implemented. While utilizing this type of systematic approach to intervention development is necessary, extending its use in reporting both successful and unsuccessful interventions is critical to creating generalizable knowledge which will lead to improved care delivery.

**Table 3:** **Overview of available reporting guidelines for the implementation of interventions**.

Reporting guideline	Method of development	Goal of guideline
Workgroup for intervention development and evaluation research (WIDER) group recommendations (31)	Expert recommendations to journal editors	Describes extensions to the CONSORT guidelines that will facilitate better communication of behavioral change interventions
Template for intervention description and replication (TIDieR) checklist (32)	Created through expansion of CONSORT criteria using a modified Delphi consensus approach	Describes a 12 item checklist to improve the completeness of reporting of interventions to improve replicability
Criteria for reporting the development and evaluation of complex interventions in health care (CReDECI2) (33)	Created through a systematic literature review and expert review	Describes a criteria list of 16 items pertaining to the reporting of the (1) development, (2) feasibility and pilot testing, and (3) introduction of an intervention and evaluation
Intervention taxonomy (ITAX) (34)	Researcher review of intervention study protocols to capture key elements of the interventions important to subsequent replication	Describes a taxonomy/catalog of key features of an intervention to consider in design, execution, and reporting
Strengthening the reporting g of observation studies in epidemiology (STROBE) statement (35)	Created during a 2-day workshop with methodologists, researchers, and journal editors	Describes a checklist of 22 items to guide reporting of observational research
Standards for quality improvement reporting excellence (SQUIRE 2.0) (36, 37)	Created with input from an expert panel with public feedback	Outlines a checklist of items to consider when reporting quality improvement studies
Standards for reporting implementation studies of complex interventions (StaRI) (38)	Created by multidisciplinary panel using an e-Delphi approach	Describes standards for reporting of implementation studies

Using theoretical models to guide intervention development, identifying active implementation strategies perceived to be feasible and important, and considering the local context in which an intervention will be implemented increase the likelihood that an intervention will be successfully implemented and sustained. To highlight further how these concepts can be applied to a contemporary clinical problem relevant to women’s health, we discuss challenges and potential solutions to the implementation and dissemination of patient decision aids, focusing specifically on a breast cancer surgery decision aid.

## Breast Cancer Surgery Decision Aids

Decision aids are a form of decisional support designed for use as an adjunct to clinical consultation and can facilitate patient-driven decision-making by clarifying and contextualizing the medical and psychological issues associated with the decision (39, 40). The Affordable Care Act promotes the routine use of decision aids to improve shared decision-making and decrease unwarranted variation in care and cost (41). Many decisions for cancer treatment require patients to consider the risks and benefits of various treatments in the context of their personal values, making them especially appropriate for application of a decision aid. Consider breast cancer surgery: as survival is equivalent for both breast conservation and mastectomy, women must weigh the increased risk of recurrence associated with breast conservation against the greater impact on body image associated with mastectomy in order to make a decision that matches their personal values. Active patient participation in this decision is essential, as it is associated with less decisional regret, more satisfaction with care, improved post-operative body image, and greater long-term quality of life (42–44). Breast cancer surgery decision aids effectively support this decision-making process by improving knowledge, decreasing decisional conflict, and facilitating communication between patients and surgeons (44–47). Unfortunately, despite their proven effectiveness and perceived ease of use, only a minority of women diagnosed annually with breast cancer receive one during the course of their care (48, 49). The current limited reach of evidence-based decision aids into the everyday care of cancer patients represents an ideal example where the application of implementation science can lead to improved delivery of cancer care.

**Figure 2:**
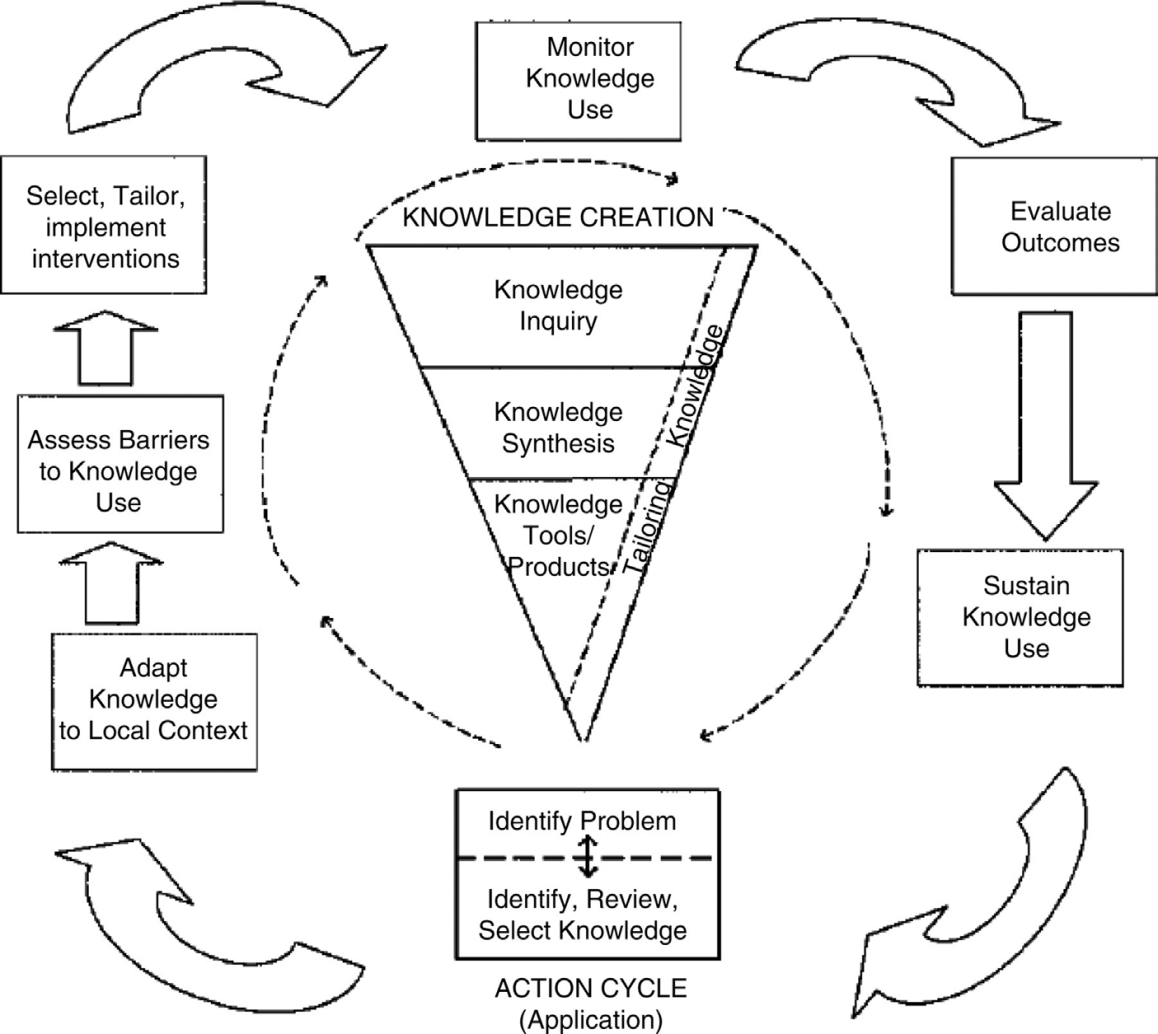
**Knowledge-to-action cycle**. From Graham et al. (50) with permission. Copyright © 2006 The Alliance for Continuing Medical Education, the Society for Medical Education, the Society for Academic Continuing Medical Education, and the Council on CME, Association for Hospital Medical Education.

A number of theoretical models could be appropriate to guide an assessment of the challenges associated with decision aid implementation and to identify a strategy for implementation that is likely to be successful. Given the wide number of available in the literature, it is more important to apply an appropriate model well, than to identify the “perfect” model. The model we will use as the example to guide our discussion surrounding the implementation of breast cancer surgery decision aids is the knowledge to action cycle (Figure 2) (50). In our example, the fundamental knowledge-to-action gap being addressed is the idea that “decision aids work, but are rarely used.” The knowledge-to-action cycle then outlines key steps to address this gap, including considering and/or adapting the intervention to the local context, assessing barriers to routine use, and selecting implementation strategies to address specific barriers.

### Adapt Knowledge to Local Context

The local context in which an intervention will be implemented has a significant influence on the success of implementation and should be considered early in the planning process (27–30). In some clinical settings, it may be advantageous to tailor the intervention or the implementation to make it more suitable for a particular population or improve the fit within an organization’s capacity. In other settings, additional implementation strategies may need to be incorporated. Adapting the implementation of an intervention to fit the local context can be an important step toward improving the success and sustainability of implementation. However, while adaptation may be desirable to maximize reach of the intervention, it is important to ensure that fidelity to the original intervention is maintained. A key step to accomplishing this is the identification of the core elements of an intervention and/or its implementation that is responsible for its effectiveness in achieving the intended outcome (9, 26, 51). The core elements can be specific components of the intervention or the specific implementation strategies essential for successful delivery of the intervention. These core elements should remain unchanged during adaptation, with tailoring focusing instead on those elements thought to be modifiable (9, 26, 51).

In our case of decision aid implementation, relevant aspects of the local context could include factors, such as financial resources of the institution, level of staffing within the specific clinic, and patient mix. These factors must be considered when developing an implementation process to ensure that implementation will be successful and sustainable. After considering these factors, examples of aspects of implementation that we would consider to be core elements critical for success would include the systematic identification of eligible patients prior to the clinical encounter (as opposed to rely on clinician identification of appropriate patients) and administration of the decision aid outside of the surgery clinical setting (as opposed to ask the clinician to administer the decision aid themselves).

### Select, Tailor, and Implement Interventions

**Figure 3:**
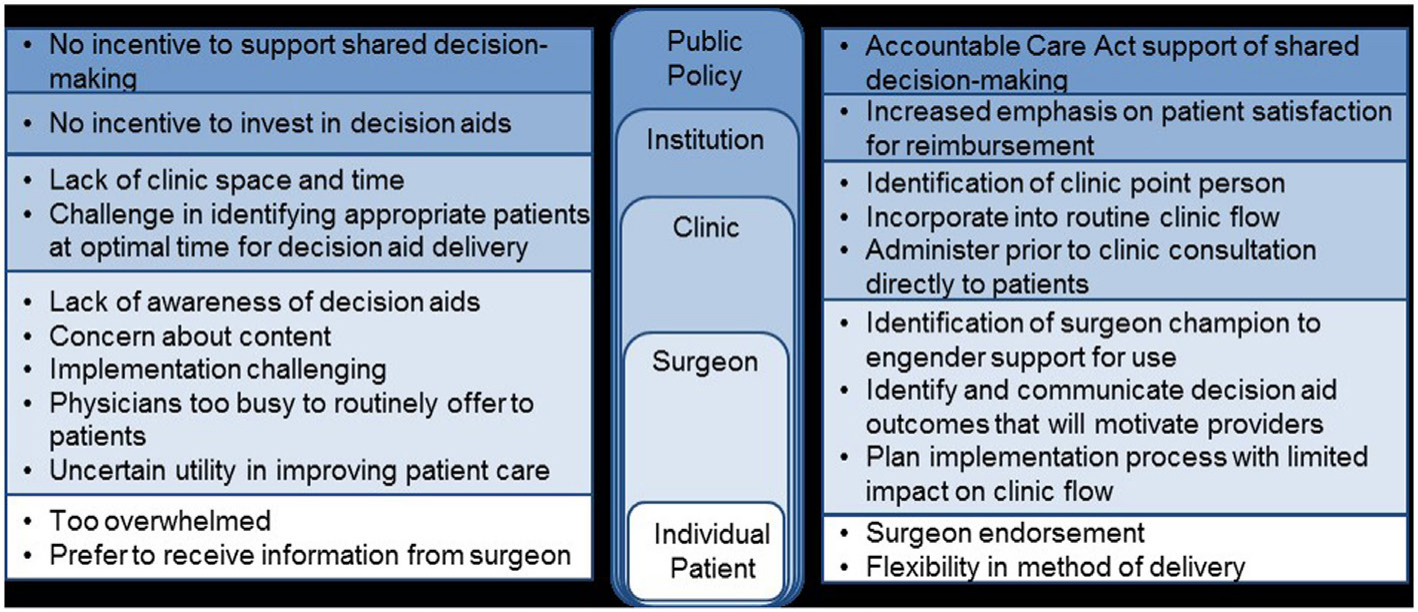
**Barriers and potential facilitators to use of a breast cancer surgery decision aid**.

Once the local context and barriers to use have been considered, implementation strategies must be selected to specifically address the known barriers. A useful tool for researchers in developing the package of implementation strategies needed is the compilation and categorization of strategies by Waltz et al. (17). In the case of decision aid implementation, we believe that “seamless” incorporation of the decision aid into routine clinical flow is critical for success. Specific challenges identified in our barrier assessment include limited clinic resources to administer decision aids, difficulty identifying appropriate patients in a timely manner, lack of surgeon buy-in, and patient preference to hear information from their surgeon (Figure 3). Although some clinics may be able to adjust their work flow to allow for decision aid administration, for many others, this is an insurmountable challenge and tailoring the logistics surrounding implementation will be necessary. One option to minimize the impact on the clinical workflow would be to utilize a decision aid administered directly to patients outside of the clinical encounter. Alternative decision aid formats, such as web-based decision aids, would be needed to accomplish this method of delivery efficiently and flexibility in method of deliver has been identified as a potential facilitator in one study (52–54). Challenges to identifying patients in a timely manner could be addressed by linking the identification of appropriate patients to scheduling of clinic visits or clinic intake calls; associating decision aid administration with a routine aspect of care already occurring will efficiently facilitate the systematic implementation of a decision aid (54). Utilization of a surgeon champion to engender support for the decision aid by other surgeons is critical for this type of intervention (54, 55). This individual can also be critical in preparing patients to be active participants in a decision aid intervention by endorsing the value of the decision aid as a way to enhance (and not subtract) from the future clinical encounter between patient and surgeon.

### Monitor Knowledge Use and Evaluate Outcomes

As identified in the knowledge-to-action cycle, monitoring use and success of an intervention over time is an important step toward sustained use (50). Evaluation models, such as RE-AIM (8) and PRECEDE–PROCEEDE (25), provide a framework for identifying relevant constructs to judge success of an implementation process. In our case example of decision aid implementation, RE-AIM would be an appropriate evaluative model, focusing on constructs, such as the ability of the implementation to *R*each all appropriate patients without introducing a systematic bias through the exclusion of certain patient populations, the *E*ffectiveness of this method of decision aid delivery as a way to improve decision quality, and the acceptability of the intervention to patients and providers as a surrogate for future *A*doption. Additional evaluative endpoints could include implementation fidelity. CFIR could also be used to evaluate implementation and explain success or lack of success (21). The various CFIR constructs can help to categorize areas where interventions fail or where specific challenges exist, and help to then identify additional potential implementation strategies. For example, in the case of decision aid implementation, if limited commitment by surgeons is identified as a barrier (characteristics of individuals construct), strategies that more strongly incorporate opinion leaders and champions could be included. If the process to implement the decision aid is perceived to be too complex for the local setting (intervention characteristics construct), adapting the implementation process to the needs of the local setting (while keeping the core elements consistent) could be explored.

A critical component of the knowledge-to-action cycle is feeding back the outcomes of these evaluations to guide iterative improvements to the implementation process. Regardless of the method utilized in monitoring the performance of the intervention, it is necessary to solicit feedback from stakeholders and actively seek out opportunities for improvement. Early identification of lapses in the implementation process can allow a timely response and create generalizable knowledge, which can inform the expansion of the intervention to other sites and clinical practices.

## Conclusion

Decreasing the gap between the identification of beneficial interventions and the incorporation of these interventions into routine clinical care is an important step toward improving the quality of cancer care delivered. Successfully addressing this gap requires a systematic and theory-driven approach to the development and subsequent implementation of interventions. The growing field of implementation science has generated, and continues to generate, a broad base of generalizable knowledge surrounding how to successfully implement and sustain interventions. As we present in our clinical example, applying the concepts of implementation science to the unique challenges associated with cancer care for women can improve the quality of the cancer care we deliver.

## Author Contributions

HN and TA were responsible for concept development, literature review, and primary manuscript writing. CG participated in concept development and review of the manuscript.

## Conflict of Interest Statement

The authors declare that the research was conducted in the absence of any commercial or financial relationships that could be construed as a potential conflict of interest.
